# Retraction: PAK3 downregulation induces cognitive impairment following cranial irradiation

**DOI:** 10.7554/eLife.110771

**Published:** 2026-01-29

**Authors:** Haksoo Lee, Hyunkoo Kang, Changjong Moon, BuHyun Youn

**Keywords:** Human, Mouse

Lee H, Kang H, Moon C, Youn B. 2023. PAK3 downregulation induces cognitive impairment following cranial irradiation. *eLife* **12**:RP89221. doi: 10.7554/eLife.89221.

Published 22 December 2023

Dr Youn, on behalf of all the authors, is retracting this eLife publication.

Concerns have been raised on PubPeer (https://pubpeer.com/publications/7E6F04806EAADD47855E7821ADCA6C) regarding the integrity of the western blot images presented in the article. Specifically, it was identified that multiple western blot bands were reused for different experimental groups and/or proteins (within and across Figures 2, 3, 4, 6 and in Figure 2—figure supplement 2). These instances involve the duplication, flipping, and contrast adjustment of image bands.

Upon review by the authors, it was determined that these figure duplications were caused by errors in data management and figure assembly during the preparation of the manuscript. Unfortunately, due to the time elapsed since the experiments were conducted, the original raw data (specifically the full-gel scans) required to validate or correct these figures are no longer available.

Given the concerns raised by these image irregularities and the inability to verify the original data, Dr Youn, in consultation with the editorial office, has decided to retract the paper to ensure the integrity of the scientific record. All of the authors agree that retraction is the appropriate course of action.

The authors apologize to the editors, reviewers, and the scientific community for any inconvenience and confusion this may have caused.

